# CONVERGENT AGING LOCI: PATHWAYS THAT TRANSCEND INDIVIDUAL DISEASES

**DOI:** 10.1093/geroni/igz038.810

**Published:** 2019-11-08

**Authors:** Luke C Pilling, Luigi Ferrucci, David Melzer

**Affiliations:** 1 University of Exeter, Exeter, United Kingdom; 2 NIA, Baltimore, Maryland, United States; 3 University of Exeter, Exeter, England, United Kingdom

## Abstract

Thousands of loci across the genome have been identified for specific diseases in genome-wide association studies (GWAS), yet very few are associated with lifespan itself. We hypothesized that specific biological pathways transcend individual diseases and affect health and lifespan more broadly. Using the published results for the most recent GWAS for 10 key age-related diseases (including coronary artery disease, type-2 diabetes, and several cancers) we identified 22 loci with a strong genetic association with at least three of the diseases. These multi-trait aging loci include known genes affecting multiple diverse health end points, such as CDKN2A/B (9p21.3) and APOE. There are also novel multi-trait genes including SH2B3 and CASC8, likely involved in hallmark pathways of aging biology, including telomere shortening and inflammation. Several of these loci involve trade-offs between chronic disease risk and cancer.

